# Philadelphia Academy of Surgery

**Published:** 1893-07

**Authors:** William J. Taylor

**Affiliations:** Surgeon to St. Agnes’ Hospital; Assistant to the Orthopædic Hospital and Infirmary for Nervous Diseases, Philadelphia, Pa.


### PHILADELPHIA ACADEMY OF SURGERY.

MEETING MARCH 6TH, 1893.

THE PRESIDENT, DR. WILLIAM HUNT, IN THE CHAIR.

### MULTIPLE FRACTURE OF BOTH UPPER EXTREMITIES.

BY WILLIAM J. TAYLOR, M. D.,

Surgeon to St. Agnes' Hospital; Assistant to the Orthopædic Hospital and Infirmary for Nervous Diseases, Philadelphia.

Margaret C., aged fifty-six years, a widow, and by occupation a monthly nurse, was admitted to my ward at St. Agnes' Hospital on the evening of October 19, 1892 suffering from the most remarkable number of fractures, considering the amount of constitutional disturbance, it has ever been my fortune to see. She was going down the cellar stairway in the dark when she missed her footing and fell to the bottom, some eight or ten steps. From the nature of the injuries she must have put out her hands before her in the hope of breaking the fall.

She was unconscious for a short time, and was then brought to the hospital in a patrol wagon, but recovered sufficiently to walk from the wagon into the receiving ward.

Upon examination it was found that she had received a lacerated wound of the scalp, six inches long and extending down to the bone, and a deep lacerated wound of the lower lip about two inches in length. There was a fracture of the surgical neck of the left humerus and an oblique fracture of the middle one-third of its shaft; a contusion of the left elbow and a fracture of the lower end of both the radius and ulna of the same side. There was a supra-condyloid fracture of the right humerus extending into the elbow-joint, forming a T. a fracture of the upper third of the radius and of the ulna, and a fracture of the lower end of the radius. In spite of this great number of fractures and of the serious lacerated wounds she was able to walk into the hospital, and seemed to suffer comparatively little pain. Her temperature was normal, her pulse good, and there was no evidence of shock such as would have been expected from the nature of her injuries.

There was much difficulty experinced in adjusting and holding in place the different fractures, but with care and patience and plenty of plaster-of-Paris this was accomplished. Her recovery has been most satisfactory, and she has for all practical purposes full use of both arms.

Such an extensive number of fractures led me to suppose there must have been some serious lesion of the bones, but the most careful inquiries failed to give me any clue to such a state of affairs. She was a large, strong, and apparently perfectly healthy woman. She had never before had a facture of a single bone, neither was there any history of fracture in any member of her family. She was born in Ireland and had lived there until a few years ago, and had always been in good health and a hard worker.

DISCUSSION.

DR. H. R. WHARTON: Some years ago the President investigated the subject of fracture of the larynx and proved that tracheotomy was indicated, and that patients usually did better after this operation.

I would ask the experience of members in regard to multiple fractures, whether they have found much constitutional disturbance or many cases of sudden death following multiple fracture. My own experience has been that generally patients do well. Last summer I had under treatment a boy six years old, who had fallen off of one of the tunnels of the B. & O. R. R., and sustained a compound fracture of the nose, fracture of both bones of each forearm, and fracture of both thighs about the middle of the shaft. The patient did perfectly well with normal temperature for a week. He was doing well when I saw him at 12 o'clock. In the evening of the same day the resident noticed that his breathing was peculiar and an hour afterward the patient was moribund. He died of some cerebral complication. I thought that it might be a case of fat embolism which is said to follow fractures. I have seen another patient die very much in the same way with simple fracture of the femur. No post-mortem was made in either case.

THE PRESIDENT : The conclusion of the paper which I wrote on fracture of the thyroid cartilage was that where emphysema and bloody sputa were present there had been up to that time no recovery where tracheotomy had not been performed. I thought that tracheotomy should be done when the first symptoms where discovered. I found several cases similar to that reported by Dr. Taylor in which recovery followed without tracheotomy.

DR. THOMAS G. MORTON : Some years ago I saw in consultation a lady, eighty-four years of age, who had gradually during ten years lost her vision from cataracts. Soon after this she sustained in a fall a fracture of both bones of the forearm, the humerus about the middle and the shaft of the femur near the great trochanter. Complete recovery from these injuries following showed such an excellent repair that six months afterward I operated upon both eyes at the same sitting. Perfect vision followed in each, which continued until her death when in her ninety-seventh year.

